# Bridging Myositis Ossificans After Supracondylar Humeral Fracture in a Child: A Case Report

**DOI:** 10.3389/fped.2021.746133

**Published:** 2021-11-18

**Authors:** Jiayuan Chen, Qilin Li, Tianjing Liu, Guoqiang Jia, Enbo Wang

**Affiliations:** ^1^Department of Pediatric Orthopedics, Shengjing Hospital of China Medical University, Shenyang, China; ^2^Department of Pediatric Orthopaedic and Trauma, Qilu Children's Hospital of Shandong University, Jinan, China; ^3^Department of Pediatric Orthopedics, Children's Hospital of Anhui Medical University, Hefei, China

**Keywords:** supracondylar humerus fracture, brachialis, myositis ossificans, operative treatment, child

## Abstract

**Background:** Myositis ossificans is an uncommon complication of trauma and surgery, defined as ossifying changes in a non-osseous tissue such as muscles. It happens after tissue injury, with or without fractures. When myositis ossificans occurs around a joint, it can cause ankylosis, leading to complete dysfunction of the joint. Though it has been described in most parts of the body, bridging myositis ossificans involving the elbow joint were scarcely reported.

**Case Presentation:** We report a severe case of myositis ossificans after a supracondylar humerus fracture in a 9-year-old child. In this case a palpable painless mass appeared following the fracture and surgical trauma. Ultrasound or X-ray is of significant diagnostic value. The brachialis was completely ossified and formed a bony bridge around the elbow, causing complete ankylosis. The bone mass was surgically removed through a bilateral less-invasive approach with less surgical trauma 9 months after initial presentation. we applied bone wax to the fresh bone wounds to prevent the formation of hematocele. Indomethacin, a non-steroidal anti-inflammatory drug, was administered after the operation to suppress bone proliferation in our case. Our patient had the best possible functional status and no recurrence at 2 years' follow-up.

**Conclusion:** Elbow myositis ossificans in children may mainly affects the brachialis. A bilateral less-invasive approach is sufficient to remove the bone mass with less surgical trauma. This case also provides a new reference for the treatment of myositis ossificans after the elbow injuries.

## Introduction

Myositis ossificans is characterized by heterotopic calcification and ossification of muscular tissue that mainly affects teenagers, but it has also been described in younger children. The disease is often limited to a single muscle and can occur throughout the body. It is associated with multiple etiologies, such as injury, genetic pre-disposition, post-infection, or undetermined causes, whereas injury is an important factor in its pathogenesis (1). The main clinical findings include limited range of motion and a palpable osseous mass. Although spontaneous resolution of myositis ossificans has been reported in up to 38% of lesions (2), excision is unavoidable in circumstances where the myositic mass limits daily activities. Myositis ossificans is a remarkably rare complication of supracondylar fractures, with two similar cases previously reported (3–8). We discussed the involved muscle, operation techniques and post-operative management comparing with two similar cases reported in the literature.

This study was approved by the Research and Ethics Committee of our institution, and written informed consent was obtained from the patient's family.

## Case Presentation

**Figure 1:**
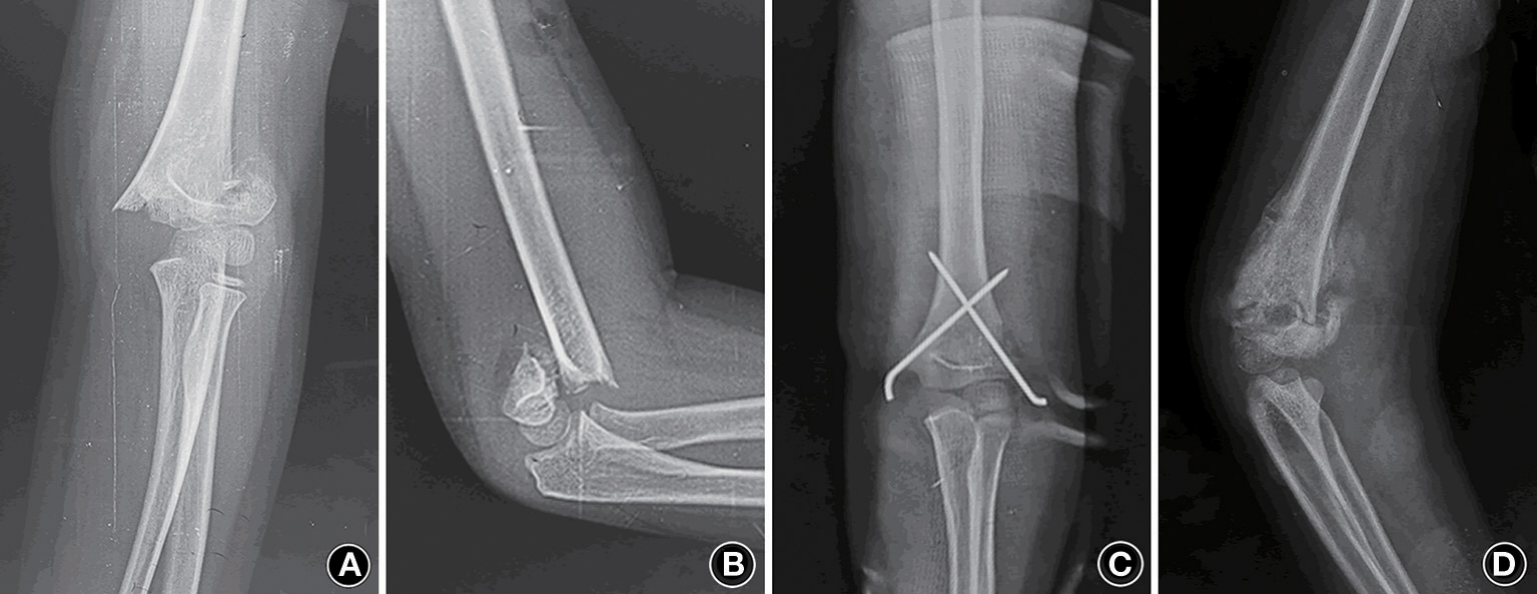
**(A,B)** Anteroposterior and lateral view of the left elbow present a supracondylar fracture of the humerus (Gartland type III). **(C)** Anteroposterior radiograph after the first surgery shows the fracture reduced with cross pinning. **(D)** Radiograph of the left elbow show some calcificated tissue around the elbow 3 months after the first operation.

**Figure 2:**
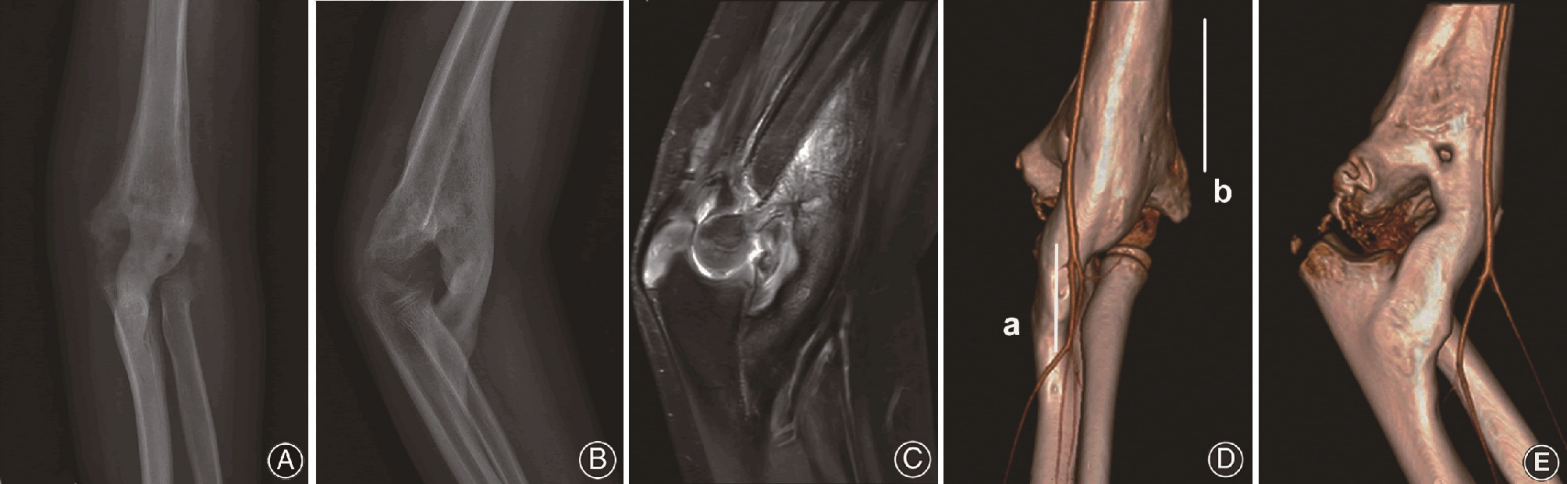
**(A,B)** Radiograph of the left elbow depicts the bridging myositis over the elbow 9 months after the primary injury. **(C)** MRI presents the bony myositis between distal humerus and proximal ulna with a smooth elbow joint. **(D,E)** CTA show brachial artery and the medial artery passing around the bony bridge. Line a and b represent longitudinal incisions along the lateral distal humerus and the medial proximal ulna respectively.

A 9-year-old child presented to the rural hospital complaining of left elbow pain after a fall. X-ray showed supracondylar fracture of the humerus classified as Gartland type III (Figures 1A,B). An open reduction was performed by the orthopedic surgeon for adult through the postcubital approach and the fracture was fixed with one lateral and one medial pin (Figure 1C). After surgery the elbow was immobilized in an above-elbow posterior splint with the elbow at 80 degrees of flexion and the forearm in neutral position. The cast and the pins were removed 6 weeks after the operation. Active and gentle passive rehabilitation practice started 1 week later and lasted 2–3 h a day. The elbow became swollen on the tenth day of rehabilitation, and the X-ray taken on the same day showed some tissue calcificated around the elbow joint (Figure 1D). The practice went on and the limited range on elbow motion aggravated, and so was the calcificated tissue (Figures 2A,B). Five months later the elbow was totally fixed at 40 degrees of flexion (**Figure 4A**).

The patient was then transferred to a territory pediatric orthopedic center 9 months after the primary injury. Laboratory tests, magnetic resonance imaging (MRI) (Figure 2C) and computed tomography angiography (CTA) (Figures 2D,E)were performed. The diagnosis of myositis ossificans was made and surgical removal of the bone bridge was planned. The surgery was performed under general anesthesia with the patient in the supine position and a pneumatic tourniquet was applied. Two longitudinal incisions were made. One was a 6-cm incision at the posterolateral part of the elbow. Enter between the triceps and the origins of the extensor carpi radialis longus and brachioradialis. Expose the humeral end of the myositic mass from the level of coronoid fossa toward the proximal part. Carefully avoid the radial nerve where it entered the interval between the brachialis and brachioradialis muscles. Strip the humeral end of the bone bridge from the anterior cortex of the distal humerus together with the calus in the radial and coronoid fossa.

**Figure 3:**
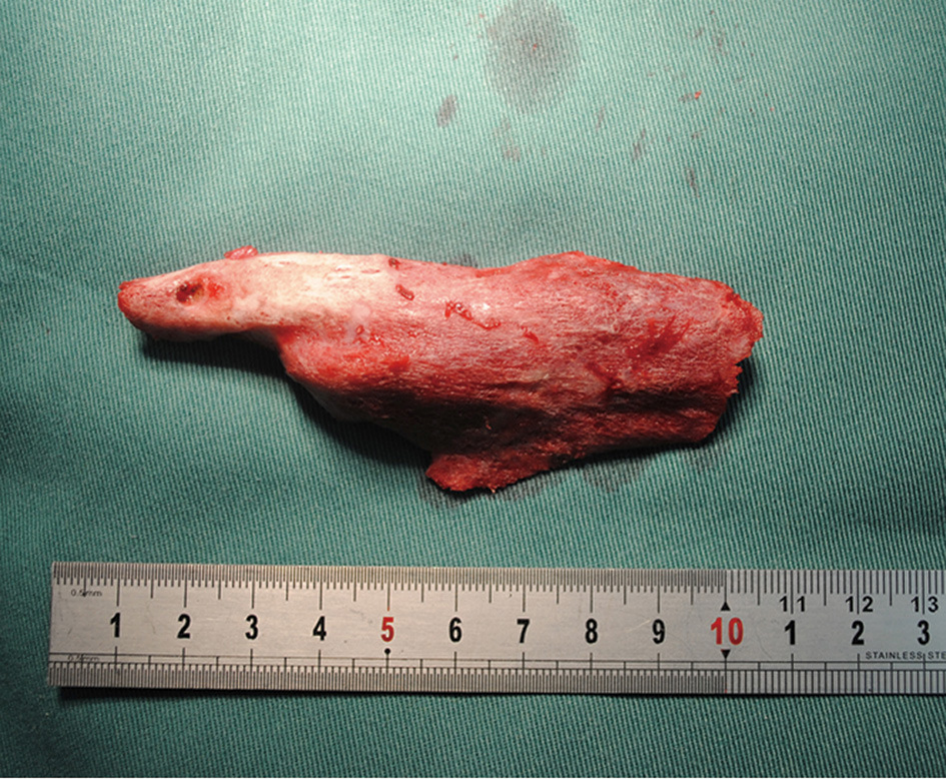
Bony myositic mass excised is about 9 × 2 cm.

**Figure 4:**
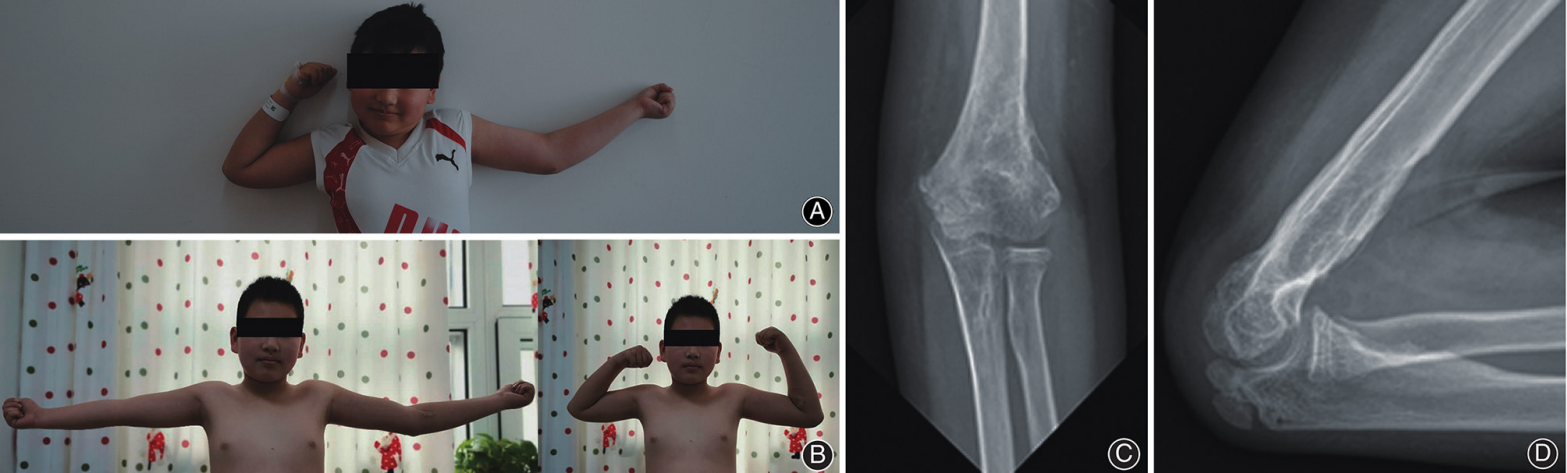
**(A)** Pre-operative photos shows a 40 degrees rigid flexion deformity of the left elbow. **(B)** Photograph of the left elbow after 2 year of the second operation shows a range of motion from 20 to 120 degrees. **(C,D)** Anteroposterior and maximum-flexion lateral radiograph of the elbow suggest no signs of recurrence.

Then a 5-cm longitudinal incision at the antero-medial part of the forearm was made. Develop the incision through the aponeurosis of the biceps. Expose the volar side of the proximal ulna through the interval between the pronator teres and the neurovascular bundle (the median nerve and the ulnar artery). The ulnar end of the bony bridge was shown and stripped from the ulna with osteotome. By this way the bone bridge was completely dissected from the bases and extracted from the humeral incision after thorough blunt dissection of the middle part (Figure 3). The mass was 9 × 2 cm in size. Bone wax was applied to the bone wound in an effort to avoid post-operative bleeding. Indometacin was used for 2 months trying to prevent recurrence. Continuous passive motion (CPM) started right after the surgery. The patient was dismissed 2 weeks later, at which time the CPM stopped and active functional exercise started. Then he was followed regularly. Two-year after operation, the range of motion was significantly improved to about 100 degrees (Figure 4B). Radiographs showed no recurrence of bone bridge (Figures 4C,D).

## Discussion and Conclusions

Myositis ossificans is an uncommon complication of upper limb trauma (8). Its pathophysiology is unclear, but muscle injury was considered a main cause of myositis ossificans (9–12). In case of local hypoxia, mesenchymal cells rapidly proliferate and differentiate into osteoblasts and osteoblasts to form bony or cartilage tissue nearby. In addition, muscle damage stimulates the production of prostaglandins, which recruit inflammatory cells to the site of injury and promote ectopic bone formation (13). Global injuries that cause extensive activation of prostaglandins may also induce ectopic bone formation at a specific site. Tetanus, head injury, hemophilia, burns, infection, neuromuscular disorders and drug abuse are risk factors of myositis ossificans (4).

Myositis ossificans after supracondylar humerus fracture were rare (14). To the best of our knowledge, there have been two similar cases reported (7, 8). One case was reported by Naranje S et al. (7). A 6-year-old boy experienced a Gartland type II supracondylar humerus fracture and was treated by close reduction and fixed with a plaster slab. Three weeks later soft tissue calcification was found in the elbow, and myositis ossificans was diagnosed after another 3 weeks. At that time the elbow developed a rigid flexion contracture of 70 degrees without any range of motion. The bone mass was surgically removed through the anterior approach 6 months after initial presentation. No measures was adopted to prevent recurrence. One year after surgery the elbow was asymptmatic with a loss of 15 degrees' range of motion.

Another case was reported by Kanthimathi B et al. (8). A 13-year-old boy had a fixed elbow contracture and a palpable bony mass in the anterior aspect of the elbow for 14 months. Ankylosis was not relieved after conservative treatment for 4 months, and finally the bone mass was removed through an anterior approach. Post-operatively the patient presented some symptoms of medial nerve injury, but it recovered spontaneously in 2 weeks after operation. The function of the elbow fully recovered within 10 weeks after surgery.

The patient in our case received open reduction and crossed pin fixation, while the two cases in literature were treated with external fixation only. Our case was initially treated by the orthopedic surgeon for adult with unprofessional rehabilitation in fear of elbow stiffness. On the contrary, this might significantly increase the risk of developing myositis ossificans (15). Although the chance of spontaneous healing of myositis ossificans might not be low, this should be weighed against the inconvenience and complications caused by long-term elbow dysfunction, such as elbow stiffness and difficulties in personal hygiene (16, 17). Local hemorrhage might also contribute to the incidence of myositis ossificans, so we applied bone wax to the fresh bone wounds to prevent the formation of hematocele. Indomethacin, a non-steroidal anti-inflammatory drug, was administered after removal of the bone mass to suppress bone proliferation in our case, but the other two cases did not use any medication. The literature has reported that low dose radiation, the use of etidronate sodium or non-steroidal anti-inflammatory drugs (NSAIDs) can help prevent recurrence (18, 19), but they seemed not indispensable. Note that the ectopic cancellous bone in the coronal fossa and radial fossa should be removed thoroughly and the bony wound be covered with bone wax in order to achieve the optimal elbow flexion.

Our intraoperative findings confirmed the involvement of the entire brachialis. The brachialis arises from the anterior inferior part of the humerus and inserts at the ulnar tuberosity and coronal process, which was exactly the starting and ending point of the bone bridge in our case. The brachial artery travels anterolaterally to the brachialis. The ossified tissue in other two cases had similar shape, so it can be inferred that the site of ossifying myositis is also the brachialis. As inferred from our case, the distal fragment normally displaces posteriorly in relation to the proximal fragment in humeral supracondylar fractures (the extension type), so that the brachialis would easily be injured by the sharp end of the proximal fragment. This injury pre-disposes the brachialis to ectopic ossification. Besides, the brachialis might be extensively stretched during rehabilitation, adding to the risk of further injury. Therefore, we suggest that anterior myositis ossificans of the elbow with brachial involvement may be a common form of myositis ossificans in children.

Based on the anatomy of brachialis and the location of the ectopic bone mass, we proposed a modified bilaterally co-approach for the removal of the bone bridge. As the middle part of the mass was not connected with the humerus or ulna, the starting and ending points of the bone bridge can be determined according to the pre-operative and intraoperative imaging examination. A longitudinal incision at both ends of the mass was made to expose the ends of the mass, and the bone bridge was dissected at the base of the conjunctions with the humerus and the ulna. After careful blunt dissection of the entire bone tissue next to the bundle of artery and the median nerve, it can be extracted from the humeral incision freely (Figure 3). This modified operation had less trauma and scar, which might be beneficial to the recovery of elbow function.

In conclusion, we reported a rare case of myositis ossificans after supracondylar fracture of humerus. For these similar three cases, myositis ossificans occurs 3 weeks−3 months after injury. It could occur after conservative fixation with a simple plaster cast or even after open reduction. By comparing with similar cases in literature, we confirmed that elbow myositis ossificans in children mainly affected the brachialis. Based on the anatomic feature of the brachialis, we designed a bilateral less-invasive approach to remove the bone mass, aiming at reducing surgical trauma. The efficacy of intraoperative bone wax and post-operative NSAIDs need further evaluation.

## Data Availability Statement

The original contributions presented in the study are included in the article/supplementary material, further inquiries can be directed to the corresponding author.

## Ethics Statement

The studies involving human participants were reviewed and approved by the Research and Ethics Committee of China Medical University. Written informed consent to participate in this study was provided by the participants' legal guardian/next of kin. Written informed consent was obtained from the individual(s), and minor(s)' legal guardian/next of kin, for the publication of any potentially identifiable images or data included in this article.

## Author Contributions

EW, TL, QL, and JC participated in diagnosing and treating the patient and acquisition of data. JC and QL wrote the case report including performing the literature review. EW approved the final version of the manuscript. All authors participated in every revision and improvement of the manuscript and read and approved the final manuscript.

## Conflict of Interest

The authors declare that the research was conducted in the absence of any commercial or financial relationships that could be construed as a potential conflict of interest.

## Publisher's Note

All claims expressed in this article are solely those of the authors and do not necessarily represent those of their affiliated organizations, or those of the publisher, the editors and the reviewers. Any product that may be evaluated in this article, or claim that may be made by its manufacturer, is not guaranteed or endorsed by the publisher.

## Abbreviations

CT: computed tomography
CTA: computed tomography angiography
MRI: magnetic resonance imaging.
